# Integrating Gut Microbiota and Violence Exposure Metrics to Classify Psychological Distress in Middle-Aged Adults

**DOI:** 10.1093/geroni/igaf122.648

**Published:** 2025-12-31

**Authors:** Khalil Iktilat, Gali Levin, Sondra Turjeman, Roy Tzemah-Shahar, Yoram Louzoun, Omry Koren, Maayan Agmon

**Affiliations:** Tel Aviv University, Tel Aviv, Tel Aviv, Israel; Bar-Ilan University, Ramat Gan, HaMerkaz, Israel; Bar-Ilan University, Ramat Gan, HaMerkaz, Israel; University of Haifa, Haifa, HaZafon, Israel; Bar-Ilan University, Ramat Gan, HaMerkaz, Israel; Bar-Ilan University, Ramat Gan, HaMerkaz, Israel; University of Haifa, Haifa, HaZafon, Israel

## Abstract

The link between violence exposure and psychological distress is well established. The study of microbiota-gut-brain crosstalk shows that the relationships between microbiota and environmental stressors are bidirectional and play a causative role in host mental state. We explored how the microbiota in a population at the onset of aging relates to violence exposure and distress levels and determined that the microbiota and exposure to violence can be used to classify patients into high- and low-distress groups. Bioinformatics and modeling were completed, underscoring the significant impact of the microbiome on violence-distress relationships and highlighting the importance of our contribution to ongoing research. We first characterized fecal microbiota of Israeli-Muslims (n = 305) aged 50.82±6.94 (40-65yrs) exposed to increasing violence in the past decade and examined correlations with exposure to violence (Screen for Adolescent Violence Exposure (SAVE) questionnaire) and psychological distress (Kessler 6) focusing on total microbiome and a subset of taxa identified from literature searches. We found unique microbial signatures associated with increasing violence exposure and distress. While some significantly associated bacteria were previously identified, many correlations were novel such as Desulfovibrio. Furthermore, microbial profiles associated with violence and distress were largely non-overlapping, yet we could classify participants into high- and low distress categories with machine learning algorithms (area-under-the-curve=0.63). This research highlights the significant role of the microbiota-gut-brain axis in linking violence exposure to psychological distress among middle-aged Muslims in Israel. It suggests that specific gut microbiota profiles, correlated with violence exposure, can help identify individuals at higher risk for psychological distress.

